# Radiation Response of Cervical Cancer Stem Cells Is Associated with Pretreatment Proportion of These Cells and Physical Status of HPV DNA

**DOI:** 10.3390/ijms22031445

**Published:** 2021-02-01

**Authors:** Irina Zamulaeva, Elena Selivanova, Olga Matchuk, Valentina Kiseleva, Liana Mkrtchyan, Ludmila Krikunova

**Affiliations:** 1 Department of Radiation Biochemistry, A. Tsyb Medical Radiological Research Center—Branch of the National Medical Research Radiological Center of the Ministry of Health of the Russian Federation, Korolev str.-4, 249036 Obninsk, Russia; selivanova_l@mail.ru (E.S.); matchyk@mail.ru (O.M.); vaivki@mrrc.obninsk.ru (V.K.); 2 Department of Radiation and Combined Treatment of Gynecological Diseases, A. Tsyb Medical Radiological Research Center, Korolev str.-4, 249036 Obninsk, Russia; liana6969@mail.ru (L.M.); gynec@mrrc.obninsk.ru (L.K.)

**Keywords:** cervical cancer, radiotherapy, cancer stem cells, human papillomavirus, flow cytometry

## Abstract

Radio- and chemoresistance of cancer stem cells (CSCs) is considered as one of the possible causes of adverse results of chemoradiotherapy for various malignancies, including cervical cancer. However, little is known about quantitative changes in the CSC subpopulation in the course of treatment and mechanisms for individual response of CSCs to therapy. The purpose of the study was to evaluate the association of radiation response of cervical CSCs with clinical and morphological parameters of disease and features of human papillomavirus (HPV) infection. The proportion of CD44^+^CD24^low^ CSCs was determined by flow cytometry in cervical scrapings from 55 patients with squamous cell carcinoma of uterine cervix before treatment and after fractionated irradiation at a total dose of 10 Gy. Real-time PCR assay was used to evaluate molecular parameters of HPV DNA. Post-radiation increase in the CSC proportion was found in 47.3% of patients. Clinical and morphological parameters (stage, status of lymph node involvement, and histological type) were not significantly correlated with radiation changes in the CSC proportion. Single- and multifactor analyses revealed two independent indicators affecting the radiation response of CSCs: initial proportion of CSCs and physical status of HPV DNA (R = 0.86, *p* = 0.001 for the multiple regression model in the whole).

## 1. Introduction

The role of ionizing radiation in treatment of malignant neoplasms is permanently increasing owing to the high efficacy of radiotherapy accompanied by organ-preservation. Hence, radiotherapy allows to achieve favorable therapeutic outcomes and good recovery along with better social and family rehabilitation. However, it is known that the radiosensitivity of cancer cells significantly differs at individual level with the same clinical and morphological parameters of malignant neoplasms (anatomic region, stage, histological type, and degree of cancer cell differentiation). This fact makes the use of radiotherapy ineffective in some cases and requires the need to identify such patients before or at the first stages of treatment in order to optimize the radiotherapy schedule.

Radio- and chemoresistance of cancer stem cells (CSCs) is considered as one of the possible causes of adverse results of treatment for various malignancies, including cervical cancer (CC) [1,2,3]. We have previously found a wide individual variability in quantitative changes of the CSC pool after the first sessions of radiation therapy and the predictive value of radiation response of cervical CSCs has been demonstrated for short-term results of treatment (the degree of tumor regression 3–6 months after treatment) [4]. Elucidation of reasons for individual variability of CSC radiation response is of undoubted interest not only from a theoretical, but also from a practical point of view, given the predictive value of these cells.

It can be assumed that mechanisms of radiation response of cervical CSCs are associated with individual features of human papillomavirus (HPV) infection, which is the main etiological factor for cancer of this localization. The basis for this assumption is the known experimental data on the effect of HPV oncoproteins on the radiosensitivity of cancer cells [5,6] and signaling pathways associated with maintenance of stemness and formation of CSC subpopulation after exposure to ionizing radiation and chemotherapeutic drugs [7,8,9,10]. Herein, HPV infection in CC patients is characterized by significant diversity in features such as genotype, viral load, and the physical status of HPV DNA (episomal or integrated form). According to our preliminary data obtained in a relatively small sample of patients, the integration of HPV DNA into the cell genome can affect properties of CSCs and, in particular, the radiation response of this important subpopulation of cancer cells [11]. However, the possible relationship of postradiation changes in the CSC proportion with clinical and morphological parameters has not been studied.

The purpose of the study was to evaluate the association of the radiation response of cervical CSCs with clinical and morphological parameters of disease (stage, status of lymph node involvement, and histological type) and individual features of HPV infection such as genotype, viral load (the number of copies of viral DNA), and physical status of HPV DNA (absence or presence of HPV DNA integration into cell genome and degree of such integration) in a larger group of patients treated with radiation and chemoradiation therapy.

Flow cytometry, which is one of the main methods for studying CSCs, was used in our study to determine the proportion of CSCs in cervical scrapings from patients with squamous cell CC before treatment and after fractionated irradiation of the primary tumor focus at a total dose (TD) of 10 Gy, as the prognostic value of the radiation response of CSCs was previously shown for this dose [4]. The markers for the identification of cervical CSCs were selected on basis of the published data on high CD44 expression and low CD24 expression on the surface of these cells [7,12,13]. Additional staining with antibodies to CD45 was performed for negative selection of lymphoid cells and binding with Hoechst33342 was used for positive selection of nucleated cells and negative selection of debris and erythrocytes.

## 2. Results

**Figure 1:**
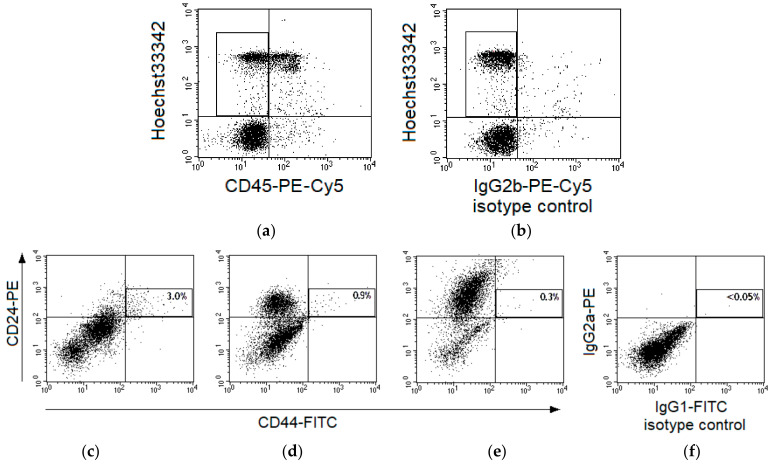
Gating strategy for identification of CD44^+^CD24^low^ cells in cervical scrapings from patients with cervical cancer (CC). Typical fluorescence of cervical cells after staining with Hoechst33342 and monoclonal antibodies labeled with phycoerythrin (PE)-Cy5 to human CD45 (**a**) or limpet hemocyanin (**b**), isotype control of nonspecific binding). The region of CD45^-^Hoechst33342^+^ events is selected for subsequent analysis. Representative dot plots for fluorescence of cervical cells in samples stained with antibodies to CD44-fluorescein isothiocyanate (FITC) and CD24-PE (**c**–**e**) or in a sample of isotype control (**f**). Cases with different expression of CD24 in the main part of cells are presented: no expression (**c**), relatively low expression (**d**), and relatively high expression (**e**). Each plot shows five thousand cells (part of the saved file for each case) after gating for CD45^-^Hoechst33342^+^ events. The region of CD44^+^CD24^low^ cells is highlighted and their percentage among CD45^-^Hoechst33342^+^ events is indicated for the entire file.

CSCs were identified as CD44^+^CD24^low^ events among CD45^-^Hoechst 33342^+^ nucleated cells in cervical scrapings of 55 patients (Figure 1a,b). The region of CD44^+^CD24^low^ cells was selected taking into account variability of CD24 expression in samples from various patients (Figure 1c–e) and isotype control of nonspecific binding (Figure 1f).

**Figure 2:**
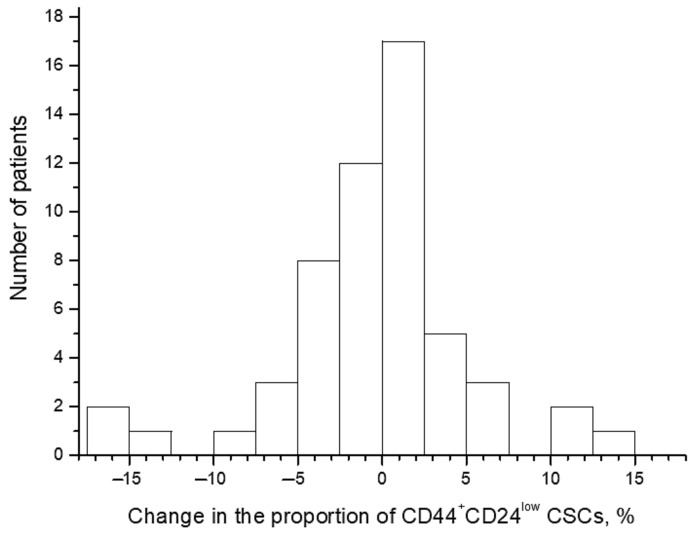
Distribution of patients with CC according to the changes in the proportion of CD44^+^CD24^low^ cancer stem cells (CSCs) after radiation exposure at a total dose (TD) of 10 Gy. The changes were calculated for each patient by the following formula: (the proportion of the CSCs after radiation exposure) − (the pretreatment CSC proportion). Thus, positive values indicate an increase in the proportion of CSCs after irradiation, whereas negative values indicate a decrease in this indicator.

**Figure 3:**
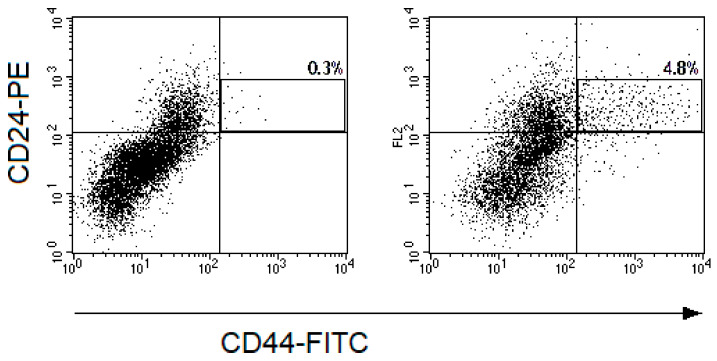
Representative dot plots for CD44-FITC and CD24-PE fluorescence of cells from cervical scrapings from the patient with CC: (**a**) before the treatment and (**b**) 24 h after irradiation at a TD of 10 Gy. The cell suspensions were stained with monoclonal antibodies to CD24-PE, CD44-FITC, CD45-PE-Cy5, and Hoechst 33342 DNA-binding dye. The gate of CD45^-^Hoechst33342^+^ events was selected and fluorescence of cells with CD44 and CD24 antibodies were evaluated in this gate. The region of CD44^+^CD24^low^ CSCs is highlighted on the dot plots presented. The percentages of CD44^+^CD24^low^ cells among CD45^-^Hoechst33342^+^ events are indicated on the plots.

The proportion of CD44^+^CD24^low^ CSCs widely varied from 0.1% to 17.1% before the treatment and from 0.1% to 19.3% after irradiation at a TD of 10 Gy. The average proportion of CSCs was 4.1 ± 0.5% before the treatment and 3.7 ± 0.5% after irradiation (*p* = 0.57). The CSC proportion increased in 26 patients (47.3%) after radiation exposure and decreased in the other 29 patients (52.7%) (Figure 2 and Figure 3). Short-term results of the treatment (complete or partial tumor regression) were known for 51 patients. Importantly, at complete tumor regression (*n* = 35), the proportion of CSCs decreased on average by 1.3 ± 0.8% after irradiation, while at partial tumor regression (*n* = 16), this indicator increased on average by 2.1 ± 1.4% (*p* = 0.04). These data confirm the predictive value of the CSC radiation response established earlier in a smaller group of 31 patients for short-term treatment results [4]. The pretreatment proportion of CSCs was not associated with tumor regression degree.

**Figure 4:**
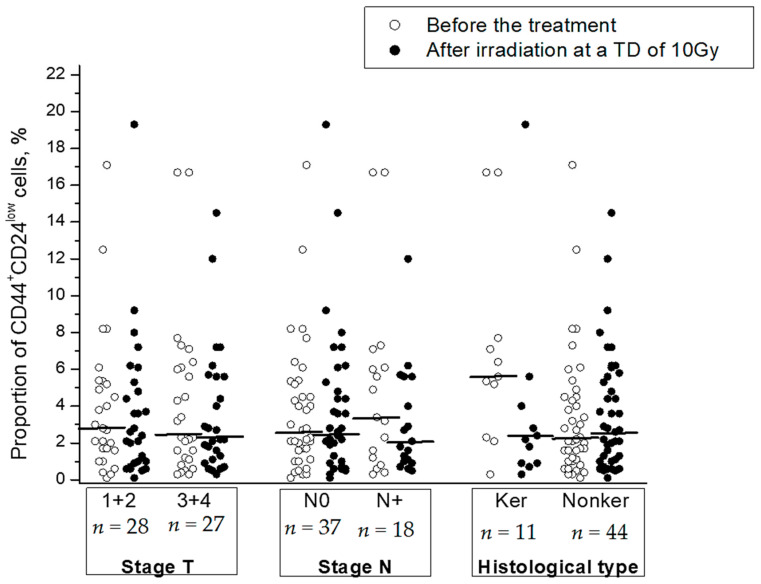
The proportion of CD44^+^CD24^low^ CSCs in cervical scrapings before the treatment and after radiation exposure at a TD of 10 Gy in subgroups of CC patients with different stages and histological types of squamous cell CC: keratinizing (Ker) vs. nonkeratinizing (Nonker). The horizontal lines indicate the median.

Clinical and morphological parameters (stage, status of lymph node involvement, and histological type) were not significantly correlated with radiation changes in the CSC proportion (Table 1). No correlation was found between these parameters and the CSC proportion before or after radiation exposure to tumors except for a higher proportion of CSCs in keratinizing squamous cell CC before the treatment in comparison with that in nonkeratinizing CC (*p* = 0.04) (Figure 4).

**Figure 5:**
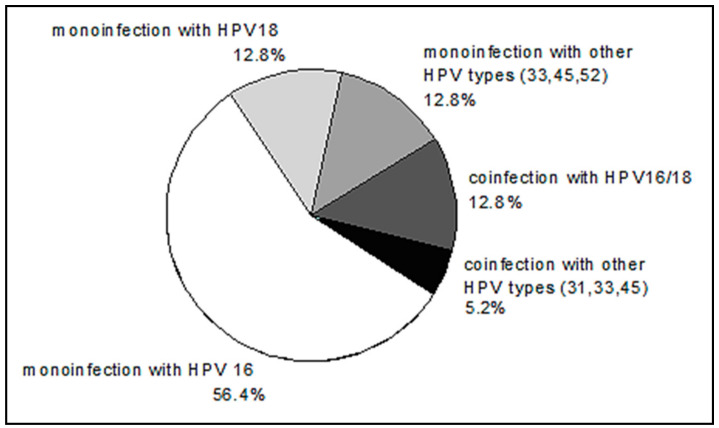
The distribution of human papillomavirus (HPV) genotypes in CC patients.

HPV DNA was not detected in two patients. The distribution of HPV genotypes in HPV-positive patients is shown in Figure 5. The distribution of HPV genotypes in our group of patients with CC is consistent with well-known data on the prevalence of HPV 16, among other genotypes.

The average number of viral DNA copies expressed as logarithm of E7 genome equivalents per 100 thousand cells was 5.6 ± 0.2. Integration of HPV DNA into the cell genome was detected in 72.2% of HPV-positive patients (average degree of integration amounted to 82.5%), whereas only the episomal form of viral DNA was found in 27.8% of patients. Molecular features of HPV infection were compared with the proportion of CSCs before the treatment and the response of this cell subpopulation to radiation exposure at a TD of 10 Gy. No parameter of HPV infection was significantly associated with the CSC proportion before the treatment or the changes in this indicator after irradiation, except for the physical status of HPV DNA, which was associated with the changes in the CSC proportion (Table 2). In particular, in tumors with fully integrated HPV DNA, the percentage of CSCs increased after irradiation (on average by 3.1%), whereas in tumors with episomal or partly integrated forms, the proportion of CSCs decreased (on average by 3.8%) (*p* = 0.03).

**Figure 6:**
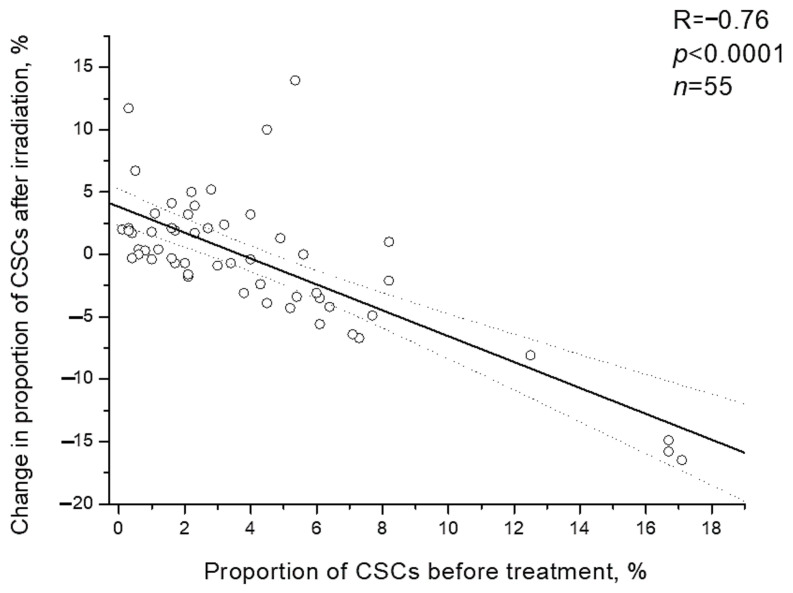
Correlation of the CSC proportion in cervical scrapings from CC patients before the treatment with change in this indicator after irradiation at a TD of 10 Gy. Dotted lines indicate 95% confidence limits for linear regression (solid line).

It should be noted that individual changes in the CSC proportion after irradiation were inversely proportional to initial proportion of CSCs before the treatment (R= −0.76; *p* < 0.0001) (Figure 6). Postradiation increase in the CSC proportion was found only in 23.8% of patients (5/21) with a high pretreatment proportion of these cells (above the average value of 4.1%) and in 64.7% of patients (22/34) with an initially low proportion of CSCs, i.e., 2.7 times more often (*p* = 0.005 according to two-tailed Fisher criterion).

Multiple regression analysis was performed to find out dependence of the changes in the CSC proportion after irradiation on possible predictors that demonstrated significant association with radiation response of CSCs in single-factor analysis (the proportion of CSCs before treatment and the physical status of HPV DNA), as well as on other parameters studied. As expected, the result revealed two independent indicators that affect the radiation change in the proportion of CSCs: the initial proportion of CSCs before treatment and the physical status of HPV DNA (Table 3). The other parameters had no predictive value, confirming the results of single-factor analysis. The constructed model was characterized by high statistical significance.

## 3. Discussion

CSCs have been detected in many types of cancer, including CC as a small subpopulation of cancer cells that possess the capacity of self-renewal and differentiation, and can drive tumor initiation, progression, and metastasis [14,15,16]. CSCs can be identified in established cancer cell lines and primary cell cultures from solid tumor tissues using available experimental methods, such as flow cytometry, immunohistochemistry, culturing in serum-free medium, and others. Flow cytometry is one of the main methods for identification and isolation of CSCs owing to the possibility of vital analysis of cell surface markers; performance of functional tests; and a high rate of data collection despite the well-known difficulties that are also inherent in other methods and are associated with heterogeneity of CSCs in expression of various markers, plasticity of this subpopulation, and lack of universal markers for CSC identification.

The overwhelming majority of flow cytometric studies of cervical CSCs were performed on established cell lines (HeLa, SiHa, and so on) [8,9,12,17,18], whereas far less studies used primary cell cultures from surgical and biopsy samples of CC [19,20,21]. As far as we know, other authors did not attempt to estimate the amount of CSCs in scrapings from cervix of CC patients by flow cytometry without preliminary culturing in vitro, which fundamentally changes the microenvironment of cancer cells and can change the expression of CSC markers depending on passage [19]. Undoubtedly, the regulation of the CSC pool in vivo before antitumor exposures and, especially, after their application, represents a significantly more sophisticated process than in cells cultured in vitro, as regulation in vivo is subject to the influence of numerous signaling molecules (for example, TGF-b1, FGF, IL-6, HIF, and Wnt ligands) released not only by tumor cells, but also by various stromal cells, including endothelial, immune cells, tumor-associated macrophages, fibroblasts, and normal stem cells [1,22]. In addition to cellular and humoral factors, the milieu conditions such as oxygen concentration and extracellular pH that affect radiosensitivity are involved in the formation of the CSC pool and the biological properties of CSCs. Therefore, the identification of patterns in the response of CSCs to radiation exposure in vivo is of great interest.

Cell surface markers (CD44, CD49f, CD90, CD133, CD271, and so on), ATP-binding cassette transporters, transcription factors (Nanog, Sox2, Oct3/4), and functional assays (side population-SP, aldehyde dehydrogenase 1) have often been used to isolate and enrich cervical CSC subpopulations by flow cytometry and sorting [14,15,23]. CD 24 is mainly used to identify breast CSCs by CD44^+^CD24^low^ immunophenotype. In CC, this marker is used less frequently, but its low expression was found to be associated with the properties of CSCs in cervical cancer cell lines [7,12,17]. Considering the literature data, we identified CSCs by CD44^+^CD24^low^ immunophenotype in cervical scrapings from CC patients. The pretreatment percentages of CSCs determined in our study were quite consistent with the range of CSC proportions found by other authors in established cell lines [8,9,12,17,18] and primary cultures [20,21] of CC using various markers and flow cytometric methods including the side population (SP) test.

Earlier, we found an increase in the proportion of cervical CSCs only in some patients after fractionated irradiation of tumors at a TD of 10 Gy [4,11], in contrast to results of in vitro studies that demonstrated a postradiation increase in the expression of markers and the proportion of CSCs in cell lines of CC and other types of cancer [12,22,24,25,26,27,28,29], because of a higher radioresistance of CSCs in comparison with that of non-stem cells and dedifferentiation of the latter under the influence of radiation. It is important to note that the increase in the proportion of CSCs after irradiation was associated with partial, but not complete, tumor regression 3–6 months after the treatment, as shown earlier and in this study in an expanded sample of patients.

In our study, the individual increase in the CSC proportion after irradiation at a TD of 10Gy was inversely correlated with the initial proportion of CSCs before the treatment. The reasons for this correlation are not fully clear. It can be assumed that, with a high initial number of CSCs, the proliferative activity of these cells is higher than in other cases, so the radiosensitivity of them is higher; therefore, the pool of these cells will decrease under the influence of ionizing radiation. On the contrary, if the number of CSCs is low, their radiosensitivity may be reduced as a result of the low proliferative activity of these cells.

The current data on association of the radiation increase in the CSC proportion with HPV DNA integration into cell genome confirmed the preliminary results obtained in a relatively small sample of CC patients [11]. On the one hand, this association appears to be due to the ability of viral oncoproteins E6 and E7 (whose expression increases as a result of integration) to influence cell cycle control, repair of DNA damage, apoptosis, and eventually induce radioresistance [5,30,31]. On the other hand, the effects of viral oncoproteins can differ in stem and non-stem cells, taking into account additional mechanisms of CSC radioresistance [1,2] and significantly higher expression of E6 oncogene in CSCs than that in other cells [7]. Moreover, enrichment of CSCs after irradiation can also be conditioned by dedifferentiation of non-stem cells as a result of radiation-induced epithelial–mesenchymal transition. It is known that viral oncoproteins are able to enhance this transition [32,33]. Taken together, an increase in the expression of viral oncoproteins after the integration of HPV DNA into cell genome, a higher oncoprotein expression in CSCs, and the ability of viral oncoproteins to increase cell radioresistance and to affect epithelial–mesenchymal transition can explain the pattern we have found regarding association of HPV DNA integration into cell genome with the increase in the CSC proportion after radiation exposure.

## 4. Materials and Methods

### 4.1. Patients and Treatment

The study group consisted of 55 patients with histologically confirmed diagnosis of squamous cell CC IB-IVA stages according to the classification developed by the International Federation of Gynecology and Obstetrics (FIGO, London, UK). This study was approved by the Ethical Committee of A. Tsyb Medical Radiological Research Center, Obninsk, Russia (protocol number 299/2018 from 1 August 2018), and all patients signed informed consent for participation in the study.

The mean age of women was 45.8 ± 1.8 years (from 23 to 70 years). All patients underwent radical courses of specialized treatment at the Department of Radiation and Combined Treatment of Gynecological Diseases (A. Tsyb Medical Radiological Research Center, Obninsk, Russia); combined radiation therapy was performed in six patients and radiochemotherapy was performed in 49 patients. The treatment in both groups of patients started with external beam irradiation of the primary tumor focus and regional metastasis zones on a linear electron accelerator SL-75-5 (Philips, Guildford, UK) in mode of photon radiation (6 MeV) fractionation at a single focal dose of 2.0 Gy daily on working days up to a TD of 30.0 Gy. Patients undergoing radiochemotherapy were administered concurrently intravenous infusions of cisplatin, 40 mg/m^2^, every week during period of external beam irradiation. Then, intracavitary radiation therapy with sources of high ^60^Co activity at a single dose of 5.0 Gy was performed two times per week until TD reached 35.0–40.0 Gy. The degree of tumor regression was determined in 3–6 months after completion of the treatment course according to the results of clinical and radiological examination (rectovaginal examination, ultrasound, magnetic resonance imaging, and so on) in accordance with RECIST recommendations (v1.1) [34].

### 4.2. Flow Cytometry

Cervical scrapings were collected before the treatment and 24 h after irradiation at a TD of 10 Gy. The material was placed in tubes containing DMEM culture medium (Paneco, Moscow, Russia) and transported to the laboratory within one hour at room temperature. Next, the cell suspension was prepared by mechanic disaggregation and filtered through a 40 μm nylon filter. Nucleated cells were counted using a Goryaev’s chamber, and 200,000–300,000 cells were aliquoted. CSCs were identified by immunophenotyping using four-color flow cytometric analysis with FACS Vantage (BD, CA, USA) equipped with two lasers (488 nm and 364 nm). The cell suspensions were stained using monoclonal antibodies labeled with different fluorochromes to the following surface markers: CD44 (clone L178 binding various CD44 isoforms), CD24, and CD45 according to the standard instructions by the manufacturer (BD, CA, USA). Hoechst 33342 DNA-binding dye (Merck, Darmstadt, Germany) was added into stained samples at a concentration of 6 µg/mL 10 min before flow cytometric analysis. The nonspecific background signal was differentiated from the specific antibody signal by isotype control using monoclonal antibodies of the corresponding isotype to limpet hemocyanin conjugated with the same fluorochromes as antibodies to the specific surface markers (BD, USA). The proportion (%) of CD44^+^CD24^low^ CSCs was estimated among CD45^-^Hoechst 33342^+^ nucleated cells, as shown in Figure 1.

### 4.3. Polymerase Chain Reaction

The parameters of HPV infection were studied in cervical scrapings before the treatment. Detection of HPV DNA of 14 high carcinogenic risk genotypes (16, 18, 31, 33, 35, 39, 45, 51, 52, 56, 58, 59, 66, 68), its genotyping, and determination of viral load were performed by real-time PCR on RotorGene (Corbett Research) using AmpliSens HPV HCR screen-titer-FL and AmpliSens HPV HCR genotype-titer FL test systems (“Central Research Institute of Epidemiology” of The Federal Service on Customers’ Rights Protection and Human Well-being Surveillance, Moscow, Russia). Integration of HPV type 16 and 18 DNA into the cell genome was detected by the TagMan method in real-time multiplex-PCR format using primers for specific amplification of viral genes E2 and E7. Sites of human β-globin gene and the specified viral genes were amplified in one test tube. At the same time, standard samples with known concentrations of HPV 16/18 DNA and β-globin DNA were amplified in each experiment, and the number of genomic equivalents of E7, E2, and β-globin was calculated from the calibration curves obtained on these standard samples. The quantitative load of HPV DNA was expressed in logarithms of E7 genomic equivalents normalized to 200 thousand genomic equivalents of human β-globin or 100 thousand cells. The degree of integration of HPV DNA was estimated by the ratio of E2 and E7 genomic equivalents, taking into account that the E7 gene remains intact during the integration of viral DNA into the cell genome, and the number of its copies in both forms of viral DNA (episomal and integrated) is the same. The E2 gene is destroyed during integration and the number of its copies is reduced.

### 4.4. Statistical Analysis

Statistical processing of the results was performed using the programs Origin 6.0 (Microcal Software, Inc., Northhampton, MA, USA) and Statistica 6.0 (StatSoft, Inc., Tulsa, OK, USA). Spearman correlation analysis was performed to assess the relationship between the two quantitative parameters. The groups were compared using the Mann–Whitney and Fisher criteria. Differences between the groups were considered statistically significant at *p* < 0.05.

## 5. Conclusions

The radiation response of CSCs is characterized by high individual variability. An increase in the CSC proportion after the first sessions of radiotherapy at a TD of 10 Gy is associated with partial, but not complete tumor regression after treatment. Single- and multifactor analyses revealed two independent indicators affecting postradiation changes in the CSC proportion; that is, the initial proportion of CSCs and the physical status of HPV DNA. Elucidation of reasons for individual variability of the CSC radiation response is of great importance for further improvement of anticancer treatment, taking into account the predictive value of this cell population.

## Figures and Tables

**Table 1:** Comparison of postradiation changes in the cancer stem cell (CSC) proportion in cervical scrapings from cervical cancer (CC) patients with different clinical and morphological parameters. FIGO, International Federation of Gynecology and Obstetrics.

Clinical and Morphological Parameters		Postradiation Changes in the CSC Proportion ^1^, %Average Value ± SE	*p*
FIGO stage	I+II	−0.3 ± 0.9	0.97
	III+IV	−0.5 ± 1.2	
Status of lymph node involvement	N0	0.2 ± 0.8	0.27
	N+	−1.6 ± 1.5	
Histological type of squamous cell CC	Keratinizing	−2.4 ± 2.6	0.23
	Nonkeratinizing	0.1 ± 0.8	

^1^ Positive values indicate an increase in the proportion of CSCs after irradiation, whereas negative values indicate a decrease in this indicator.

**Table 2:** Postradiation changes in the proportion of CD44^+^CD24^low^ CSCs in subgroups of patients with various molecular parameters of human papillomavirus (HPV) infection.

Molecular Parameters of HPV Infection		Postradiation Changes in the CSC Proportion ^1^, %Average Value *±* SE	*p*	
HPV genotype	16	−0.8 ± 1.4	0.45	
	18	−1.1 ± 0.9		
	Other genotypes (mono- or coinfection)	0.8 ± 2.0		
Viral load	Relative high (≤5.6)	−2.5 ± 1.5	0.34	
	Relatively low (>5.6)	−0.2 ± 1.9		
Physical status of HPV DNA ^2^	Absence or partial integration of HPV DNA into the cell genome	−3.8 ± 2.3	0.03	
	Full integration	3.1 ± 1.6		

^1^ Positive values indicate an increase in the proportion of CSCs after irradiation, whereas negative values indicate a decrease in this indicator. ^2^ Only for HPV 16 and 18.

**Table 3:** The results of multiple regression analysis of dependence of the changes in the CSC proportion after irradiation on possible predictors.

Indicator (Predictor)	Beta ^1^	*p* Value for Predictor	R^2^	*p* Value for Model in the Whole
The proportion of CSCs before treatment	−0.77	0.01	0.86	0.001
Physical status of HPV DNA	0.45	0.04		
Histological type	0.28	0.27		
HPV genotype	−0.01	0.96		
Viral load	0.06	0.81		
Stage T	−0.04	0.88		
Stage N	−0.05	0.85		

^1^ Beta is the standardized angular regression coefficient (in SD units). ^2^ R is the multiple correlation coefficient.

## Data Availability

The data that support the findings of this study are available from the corresponding author upon reasonable request.

## Abbreviations

CSC	cancer stem cell
HPV	human papillomavirus
PCR	polymerase chain reaction
CC	cervical cancer
TD	total dose
PE-Cy5	phycoerythrin-cyanine 5
FITC	fluorescein isothiocyanate
PE	phycoerythrin
SP	side population
FIGO	International Federation of Gynecology and Obstetrics
